# Detection and genetic characterization of Crimean-Congo hemorrhagic fever virus in ticks from western Spain (2017, 2020-2024)

**DOI:** 10.3389/fvets.2026.1789622

**Published:** 2026-04-01

**Authors:** Patricia Sánchez-Mora, Miguel A. Habela, Teresa del Peso, Ana Candela Grande Ávila, Ana María García López, Jennifer Mata García Soldado, María M. Tapia, Francisca Molero, Laura Herrero, A. Sonia Olmeda, Félix Valcárcel, Agustín Estrada-Peña, Anabel Negredo, María Paz Sánchez-Seco

**Affiliations:** 1 Centro Nacional de Microbiología, Instituto de Salud Carlos III, CNM-ISCIII, Madrid, Spain; 2 Centro de Investigación Biomédica en Red de Enfermedades Infecciosas (CIBERINFEC), Madrid, Spain; 3 Escuela de Doctorado, Universidad Autónoma de Madrid, Madrid, Spain; 4 Departamento de Sanidad Animal, Facultad de Veterinaria de Cáceres, Universidad de Extremadura, Cáceres, Spain; 5 Universidad Complutense de Madrid, Madrid, Spain; 6 Instituto Nacional de Investigación y Tecnología Agraria y Alimentaria, Madrid, Spain; 7 Facultad de Veterinaria, Universidad de Zaragoza, Zaragoza, Spain

**Keywords:** Crimean-Congo hemorrhagic fever virus, genetic diversity, *Hyalomma lusitanicum*, Spain, surveillance, viral circulation, wild ungulates

## Abstract

**Introduction:**

Crimean-Congo hemorrhagic fever virus (CCHFV) was first detected in Spain in ticks collected from red deer in southwestern Cáceres. Since then, this region, established as endemic, has been the focus of several surveillance studies. However, updated data on viral circulation in this area remain limited.

**Materials and Methods:**

We conducted a retrospective surveillance study to assess the presence and genetic diversity of CCHFV in ticks collected in central and southern Cáceres over multiple years (2017 and 2020–2024). A total of 3,183 ticks, grouped into 1,569 pools, were collected from wild ungulates, livestock, domestic animals and vegetation, and analyzed by two PCR methods. Positive pools were characterized by Sanger sequencing.

**Results:**

CCHFV was exclusively detected in *Hyalomma lusitanicum* ticks, with an overall infection rate of 1.54% (95% CI: 1.14–2.03). Most positive pools originated from wild ungulates, particularly red deer. Genetic analysis revealed the circulation of two CCHFV genotypes, predominantly genotype III.

**Discussion:**

The detection of CCHFV in ticks collected over multiple years supports the sustained circulation of the virus in southwestern Cáceres. Our findings also reinforce the key role of *H. lusitanicum* as the main vector maintaining the virus in wild ungulates and underscore the genetic diversity of circulating strains and the importance of using multiple molecular methods. These results emphasize the need for continuous surveillance in endemic areas to monitor viral circulation and assess animal and public health risks.

## Introduction

Crimean-Congo hemorrhagic fever (CCHF) is a severe tick-borne viral disease caused by CCHF virus (CCHFV), a single-stranded virus from the *Nairoviridae* family. It is considered a significant public health threat due to its high fatality rate, lack of specific treatments and safe vaccines, and its potential for nosocomial outbreaks, leading to its inclusion in the WHO R&D Blueprint (1). In humans, subclinical infections are underestimated and may represent a significant proportion of cases, despite the fatality rate can range from 5 to 40% (1).

CCHFV genome is a tripartite negative-sense RNA divided into small (S), medium (M) and large (L) segments, which are prone to reassortment (2). Its genetic diversity strongly correlates with geography, with distinct spatially segregated clades based on nucleotide similarity of the S segment (3). After the recent reclassification of genotype VI as Aigai virus, CCHFV is now classified into five different genotypes: Africa (I-III), Asia (IV), and Europe (V) (4).

Tick bites are the main transmission route for CCHFV human infections, although they can also occur through direct contact with tissues and body fluids of infected animals and individuals, including nosocomial transmission. Ticks of the genera *Hyalomma, Rhipicephalus*, and *Amblyomma variegatum* are considered proven competent vector and reservoir of this virus (5–7), even though it has also been detected in other tick species (8, 9). However, laboratory detection of tick vectorial abilities is blurred because the lack of crossed corroboration of findings (10). Infected ticks bite and transmit the virus to diverse wild and domestic animals which do not show clinical symptoms of the disease but act as amplification hosts.

CCHF is widely distributed across Africa, Europe, the Middle East and Asia, south of the parallel 50th north, which is supposed to be the geographical limit of permanent populations of the tick *Hyalomma marginatum*, the presumed main vector in the Mediterranean region (1). The first detection of CCHFV in Spain was reported in 2010 in *H. lusitanicum* ticks collected from red deer in southwestern Cáceres (11). Since then, several active surveys have been conducted in this endemic area, showing viral presence until 2017 (9, 12–14) (Supplementary Figure 1). Spain is considered one of the European countries with high probability for CCHF occurrence (15), as evidenced by the 20 human cases reported between 2013 and 2025 (16–18). Seroprevalence studies further support the role of wild boar and red deer in this region (19–21), reinforcing Cáceres as a CCHFV hot spot. The emergence of a human case in this area for the first time in 2024 supports this claim (18).

Most human cases have occurred in the large distribution area of both *H. marginatum* and *H. lusitanicum* (22). Both species are adapted to hot and arid environments, with a peak activity period from May to October (15), although climate change is expected to alter their life cycle (23). *Hyalomma lusitanicum* appears to play a key role in maintaining CCHFV in Spain (9, 12, 13), being common and abundant in the western part of the country (22), where most human cases have been reported (17, 18). This region also has an abundant population of rabbits and ungulates, the primary hosts for immatures and adults of *H. lusitanicum*, respectively. These vertebrates contribute to increasing the tick population and therefore the risk of CCHFV human infections (19, 24).

Given the spread of CCHFV in Spain in recent years and the rising of human cases, it is essential to re-examine the current viral circulation in this area. This study aims to retrospectively analyze the presence of CCHFV in ticks collected in Cáceres in 2017 and between 2020 and 2024, providing updated insights into its circulation and distribution.

## Materials and methods

### Sampling strategy

This work is a retrospective study based on the secondary use of samples collected in the context of previous projects with different primary objectives. Although the sampling was not originally designed for the current research question, it provided an opportunity to explore the presence of CCHFV in ticks in this endemic region.

Most ticks were collected during the hunting season (October-February) from 2020 to 2024, with additional ticks from 2017 (October-December). The study area was selected based on previous tick surveillance studies that had established the presence of CCHFV before (9, 11–13). Agreements with hunting estates for the tick collection determined the temporal and spatial distribution of sampling points and the primary availability of samples. As a consequence, some years and locations were sampled more intensively than others, resulting in a non-homogeneous spatial and seasonal coverage. In addition, we analyzed ticks from livestock, domestic animals and vegetation collected outside the hunting season in the same areas.

### Study area and tick collection

**Figure 1:**
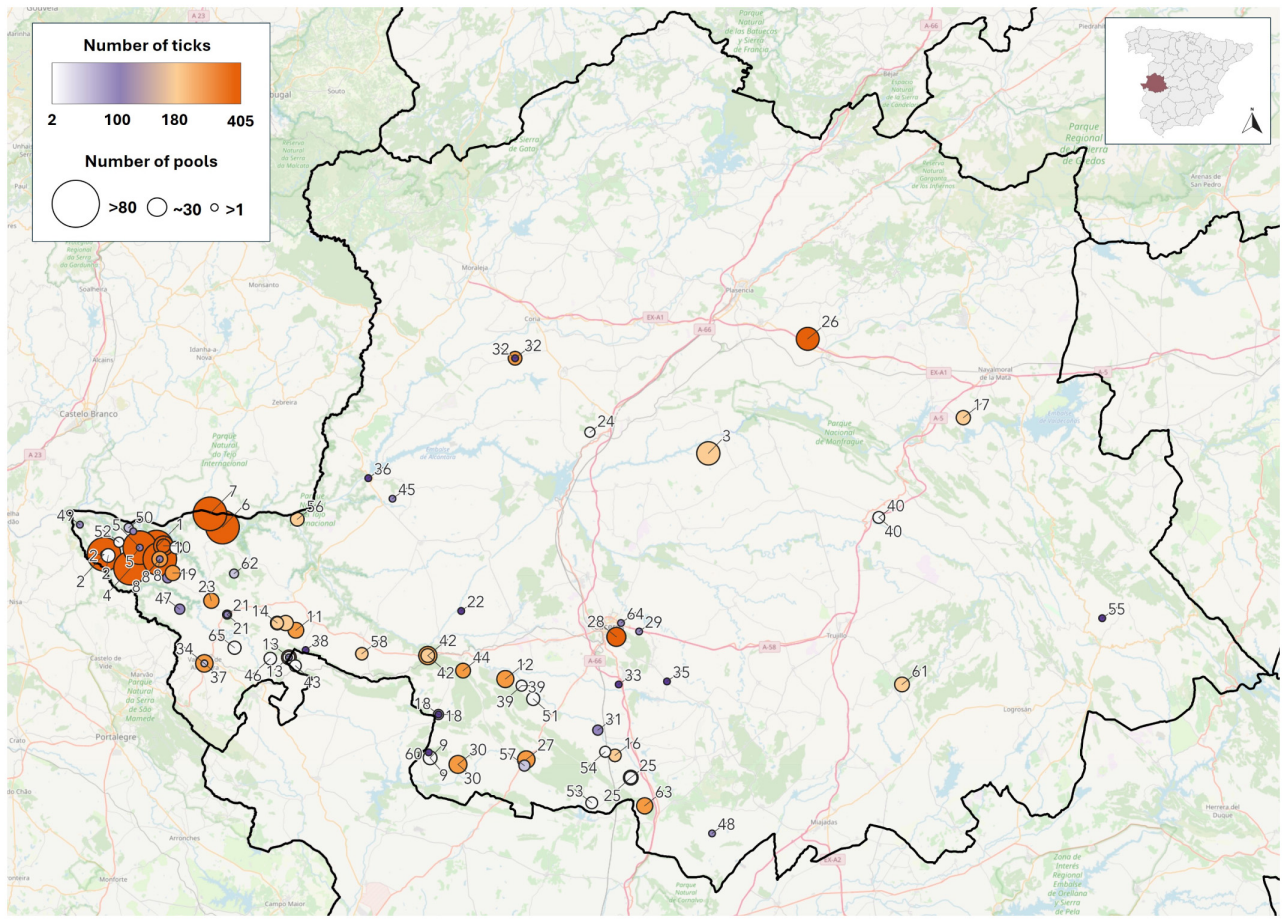
Map of Cáceres province showing all sampling points included in the study where ticks were collected between 2017 and 2024. Each point is labeled with its sampling point ID, although some overlaps occurs in areas with close proximity. Color scale represents the total number of ticks collected at each point, while the size of the circles indicates the number of pools processed at that location, as detailed in the legend.

Ticks were collected from 65 sampling points in central and southern Cáceres (western Spain) (Figure 1) during two different periods: 2017, and 2020-2024 (Supplementary Table 1).

Most ticks were obtained from wild ungulates, including red deer, wild boar, and fallow deer, as well as from livestock (cattle, ovine, and equine) and domestic animals such as dogs. These ticks were collected while feeding, taking advantage of hunts for wild ungulates or during routine management or sanitary procedures for livestock and dogs. Questing ticks were collected using the blanket dragging technique (25, 26) with a 1.5 x 2 m white towel. The blanket was dragged across the ground and/or vegetation, kept perpendicular to the direction of movement and turned over every 30 steps to collect attached ticks in a suitable container. This process was repeated for a minimum of 30 min. Further details about ticks collected at each sampling point, month and year are shown in Supplementary Table 1.

Once collected, ticks were morphologically determined to species level by trained entomologists using standard taxonomic keys. Collected ticks had well-defined morphological characters, allowing reliable species-level determination without molecular confirmation. Ticks were then sent on dry ice to the National Center of Microbiology for further processing. Before molecular analysis, ticks collected from 2020 to 2024 were grouped into pools of up to four adult individuals or up to 10 nymphs, depending on their size. A pool was always composed of specimens from the same host or point of vegetation, the same tick species, same life stage, collection date and sampling point. However, in 2017, ticks were processed and analyzed individually, each considered as single-tick pool.

### Extraction of RNA

Pools formed by whole ticks were crushed in a biosafety level 3 laboratory (BSL-3) by using plastic pestles to homogenize ticks in a mixture of 560 μL AVL buffer, included in the QIAamp Viral RNA Mini Kit (QIAGEN, Hilden, Germany) and 140 μL of RNAase-free water. After homogenization and centrifugation, supernatants were collected and transferred to 560 μL of absolute ethanol, then stored at −80 °C until the RNA extraction, carried out in a BSL-2. RNA was eluted in 60 μL of elution buffer and immediately processed.

### Molecular analysis

Two PCR assays, both based on the S segment but in non-overlapping regions, were used for the detection of CCHFV in ticks. A quantitative RT-PCR (qRT-PCR) developed by Atkinson et al. (27) modified with an internal control was performed throughout the entire study period. The second method was initially performed as a nested RT-PCR, described by Negredo et al. (28), but was later optimized into a qRT-PCR format. This optimized version was applied to the remaining pools and is described in the following section. The number of pools analyzed with each method is detailed in Supplementary Table 2.

Pools were considered positive when amplification was obtained in at least two different PCR assays designed in different genomic regions, or when a single positive result was subsequently confirmed by sequencing. All positive samples, regardless of the detection method, were subjected to the nested RT-PCR (28) to obtain a fragment for sequencing. For pools that yielded only one positive qRT-PCR result (Ct < 35) for which the sequence could not be obtained, positivity was confirmed through alternative in-house molecular methods available in the laboratory.

#### Design of the quantitative real-time PCR assay

To adapt the nested PCR step into a qRT-PCR while maintaining its original sensitivity and specificity, we designed a probe by comparing and aligning sequences available in the NCBI GenBank database (RRID:SCR_002760) from all CCHFV genotypes. The pair of primers used for this qRT-PCR were the same used in the nested PCR of the Negredo et al. (28) method. The primers/probe are listed in Table 1.

**Table 1:** Primer and probe sequences of the adapted PCR.

Primers/ Probe	Sequence (5'- 3')	Genome position ^b^
Cricon 2F+ ^a^	ARTGGAGRAARGAYATWGGYTTYCG	450–474
Cricon 2Re- ^a^	CYTTGAYRAAYTCYCTRCACCABTC	650–674
CRIC probe	6-FAM - ATGYTDTCDGAYATGA - NFQ_MGB	560–575

^a^ This pair of primers is the same used by Negredo et al. (28).

^b^ Genome position based on IbAr10200 strain (accession number: NC_005302).

The qRT-PCR was performed using the SuperScriptTM III PlatinumTM One Step RT-qPCR Kit (Thermo Fisher Scientific, USA) on a QuantStudio 5 system (Applied Biosystems, USA). The cycling conditions used were as follows: 50 °C for 30 min (reverse transcription), 95 °C for 15 s, followed by 45 cycles at 95 °C for 15 s, 53 °C for 30 s (collection of fluorescence data) and 68 °C for 30 s, with a final cooling step at 40 °C for 30 s.

The validation of this PCR assay was carried out by using a panel of previously characterized RNA extracts, including CCHFV-positive nucleic acids extracts from tick pools (*n* = 21) and nucleic acids extracts from anonymized positive human samples available in the laboratory and obtained through surveillance programs (*n* = 8), representing genotypes III, IV, and V. A set of negative tick samples was also included. These were used to compare between both types of methods, demonstrating a strong correlation between both PCRs. The optimized qRT-PCR provided high sensitivity, allowing easier and reliable processing.

### DNA sequencing and phylogenetic analysis

The amplified fragments from the nested PCR of positive samples (225 bp) were sequenced using the Sanger chain-termination method. The sequencing primers were Cricon 2F+ and Cricon 2Re–, the same used in the nested PCR (28). Consensus sequences for each segment were assembled and analyzed using the SeqMan program (DNASTAR, USA).

Phylogenetic trees were generated by using the MEGAX (Molecular Evolutionary Genetics Analysis, Version 10) program (29). The phylogenetic tree was built using the Neighbor-Joining method based on partial (127 bp) sequences of the S segment of the virus. The bootstrap consensus tree inferred from 1,000 replicates and values < 40 are not shown. The evolutionary distances were computed using the p-distance method and are in the units of the number of base differences per site.

Identical sequences were collapsed into representative groups for the tree construction. The composition of each group, including sampling year, sampling point, and GenBank accession number (if available), is provided in Supplementary Table 3.

### Data analysis

All analyses were conducted at pool level. CCHFV infection rate was estimated using the Minimum Infection Rate (MIR) parameter, calculated assuming one infected tick per positive pool, as: MIR = (number of positive pools/total ticks) × 100 (30).

To evaluate whether host identity influenced viral detection in feeding ticks, we fitted a generalized linear mixed-effects model (GLMM) with binomial error distribution and logit link function, including host type as a random effect to account for the non-independence of pools originated from the same host.

## Results

**Figure 2:**
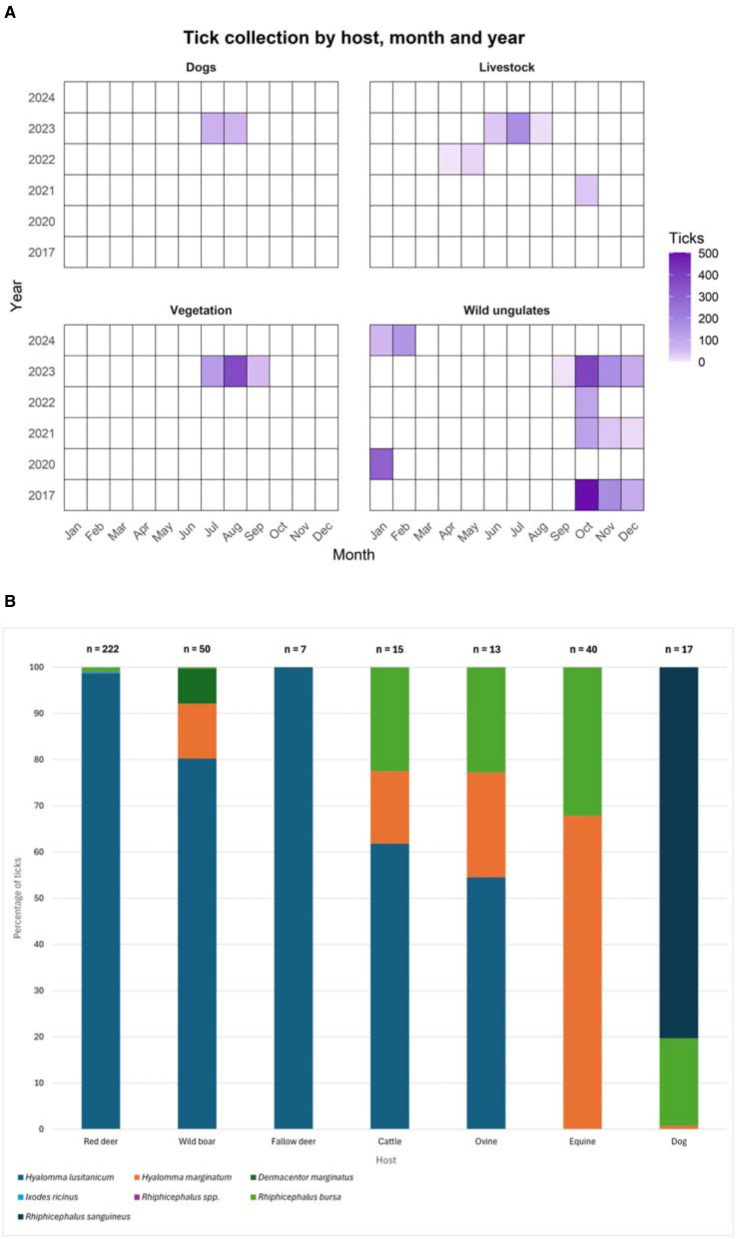
Temporal and host related patterns of the tick collection. **(A)** Heatmap of the number of ticks collected by host/vegetation, month and year of the study period (2017, 2020–2024) in Cáceres. Darker purple indicates higher number of ticks; white = no ticks available for the study; **(B)** Percentage of tick species collected from each animal host during the study period (2017, 2020–2024) in Cáceres. The number of animals is indicated above its correspondent bar.

A total of 3,183 ticks were collected in 6 years (2017; 2020–2024). Adult ticks represented the majority of the collection (*n* = 2,661; 83.6%), although nymphs were also collected (*n* = 522; 16.4%). Most ticks were obtained from wild ungulate species (red deer, wild boar and fallow deer), with additional collections from cattle, ovine and equines, as well as from dogs (Supplementary Table 1). Detailed information on the temporal distribution of collected ticks by host, month, and year is shown in Figure 2A. For molecular analysis, ticks were grouped into 1,569 pools (1,508 pools of adult ticks and 61 pools of nymphs).

*Hyalomma lusitanicum* was the most prevalent species (84.2% of the collection). Other species were detected at much lower frequencies, including *Rhipicephalus sanguineus* s.l, *H. marginatum, Rhipicephalus bursa, Dermacentor marginatus* and *Ixodes ricinus*. Only one tick could not be classified at species level but was identified within the genus *Rhipicephalus* (Table 2).

**Table 2:** Tick collection data by species and year (2017, 2020–2024).

Species	2017	2020	2021	2022	2023	2024	Total
*Hyalomma lusitanicum*	24/767 (767)	3/102 (305)	1/68 (191)	0/41 (102)	21/314 (1,146)	0/66 (170)	49/1,358 (2,681)
*Hyalomma marginatum*	–	–	0/1 (2)	0/3 (3)	0/64 (126)	0/14 (38)	0/82 (169)
*Dermacentor marginatus*	0/1 (1)	0/1 (1)	0/8 (12)	0/4 (8)	0/3 (3)	–	0/17 (25)
*Ixodes ricinus*	–	–	–	–	0/3 (3)	0/1 (2)	0/4 (5)
*Rhiphicephalus bursa*	–	–	0/1 (1)	0/11 (20)	0/40 (107)	0/1 (2)	0/53 (130)
*Rhiphicephalus sanguineus* s.l.	–	–	–	–	0/53 (172)	–	0/53 (172)
*Rhiphicephalus* spp.	–	–	–	–	0/1 (1)	–	0/1 (1)
Total	24/768 (768)	3/103 (306)	1/78 (206)	0/59 (133)	21/478 (1,558)	0/82 (212)	49/1,569 (3,183)
MIR (%) 95% CI	–	–	–	–	–	–	1.54 (1.14–2.03)

Data are shown as the number of CCHFV positive pools over the total number of pools; the number of ticks included in those pools is shown between brackets. Overall Minimum Infection Rate (MIR, %) for the entire study period is shown, calculated as the number of positive pools divided by the total of ticks x 100. The last row indicates the 95 % confidence intervals (95% CI) that were calculated by the Clopper-Pearson method (48).

“–” means no ticks of that species were collected that year.

A total of 2,632 ticks (1,503 pools) were collected from animals. The distribution of tick species varied across the different hosts (Figure 2B). *Hyalomma lusitanicum* was the predominant species, particularly in red deer, while *H. marginatum* was predominantly collected from equine livestock. Other species, such as *R. sanguineus* s.l., were only found in dogs. In addition, 551 ticks (66 pools), most of them nymphs (50/66), were collected from vegetation (Supplementary Table 1).

Of the 1,569 pools analyzed, 49 tested positive for CCHFV (Table 2). Positive pools were detected in four of the 6 years of the study period, with an overall MIR of 1.54% (95% CI: 1.14– 2.03; see Materials and Methods for calculation) (Table 2). Among the 65 sampling points included in the study area, 7 yielded positive results (Supplementary Figure 2), all of them located in rural areas of the study region characterized by Mediterranean forests and high densities of wild ungulates.

No CCHFV-positive pools were detected among questing ticks. All CCHFV-positive pools were composed of *H. lusitanicum* adult ticks collected on wild ungulates. Of the 49 CCHFV-positive pools, 44 (89.8%) were obtained from red deer and five (10.2%) from wild boar. In some cases, positive ticks were collected from one single animal among all the hosts sampled at that location and date: *H. lusitanicum* positive ticks were collected from 1 out of 23 and 1 out of 6 red deer analyzed at sampling points SP1 and SP19, respectively, as shown in Supplementary Figure 2. A GLMM was fitted to evaluate the association between host type and pool positivity. However, insufficient data in multiple host categories prevented the model for producing stable parameter estimates for all groups and it failed to converge. Therefore, the model results were not considered reliable and are not interpreted further.

CCHFV-positive pools were detected using different methods, but not all techniques yielded the same results. Of the 49 positive pools, 35 were detected by both methods, while the remaining 14 were only detected by one of the PCRs (Supplementary Table 4). Pools were classified as positive according to the criteria described in Materials and Methods.

**Figure 3:**
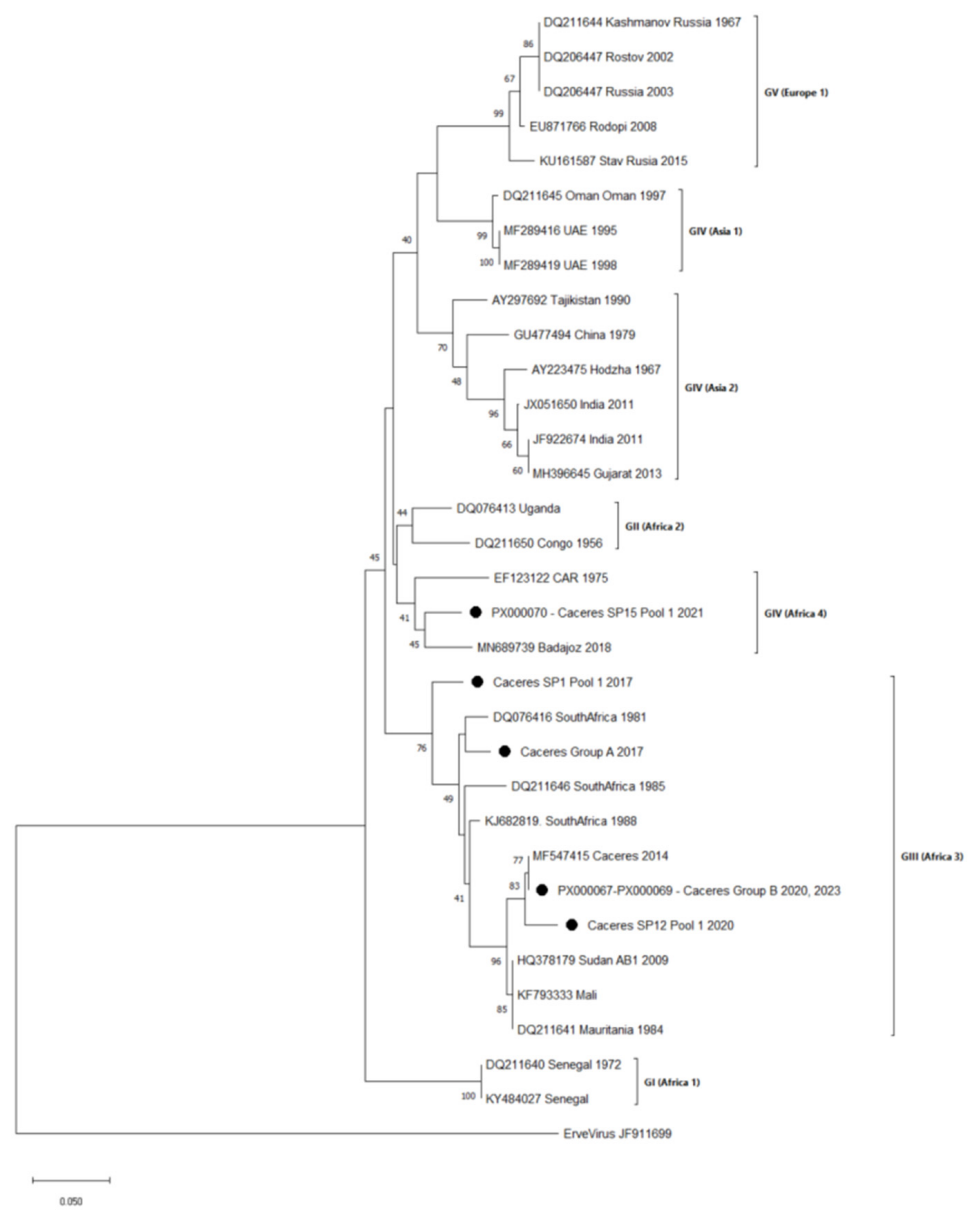
Phylogenetic tree with Crimean-Congo hemorrhagic fever virus sequences, including the sequenced strains on the S segment (127 bp) detected in this study period (2017, 2020–2024) and an additional Erve virus sequence as an outgroup. Dots indicate newly sequenced strains from Cáceres reported in this study, which are identified by GenBank accession number (if submitted), sampling point number, pool ID and year. Identical sequences were collapsed into representative groups. The composition of each group is detailed in Supplementary Table 3. Reference sequences are indicated by GenBank accession number, geographic origin, and sampling year (if known). Genotypes are indicated in roman numerals according to Carroll et al. (46). Equivalent clade nomenclature is also indicated according to Chamberlain et al. (47).

We obtained sequences from 44 out of 49 CCHFV-positive pools. Of these, 43 (97.7%) clustered within genotype III (Africa 3), while one sequence grouped into genotype IV (Africa 4). Among genotype III sequences, we observed intra-genotypic variability: strains from 2023 showed greater nucleotide similarity to those from 2020, while CCHFV strains from 2017 are clearly differentiated in a distinct group (Figure 3). The single genotype IV sequence corresponded to positive ticks collected in 2021 and clustered with a sequence obtained in 2018 from a Spanish human case (31) (Figure 3).

## Discussion

First identified in 2010 in southwestern Cáceres province (11), CCHFV has become a public health concern in Spain. In recent years, both the number of reported CCHF human cases and the CCHFV geographical range have increased in the Iberian Peninsula. The first detection of confirmed human cases in 2024 in three different Spanish provinces and in neighboring areas of Portugal (10) where no cases had previously been reported reflects this expansion and reinforces the need for continued surveillance. The risk of sporadic cases is considered moderate in areas where *Hyalomma* ticks are present, especially among people frequently exposed to tick bites (32).

*Hyalomma lusitanicum* and vertebrates like red deer and wild boar seem to be key in the enzootic maintenance of the virus in Spain (9, 12, 13). Therefore, active tick surveillance studies can provide insights into virus circulation to implement preventive measures. In this context, our study aimed to evaluate the presence of CCHFV in ticks collected in a wide area of Cáceres over multiple years (2017, 2020–2024), either feeding on hosts or questing. Feeding ticks act as sentinels of the infection, since they can be infected by feeding on a viremic host.

CCHFV was detected across multiple years of the study, supporting the endemic role of the region. The overall minimum infection rate (MIR) was 1.54% (95% CI: 1.14–2.03), consistent with previous tick surveillance studies in the same province: 1.5% by Sánchez-Seco et al. (9) using pooled samples, and 2.78% by Negredo et al. (12), based on individual tick testing. Despite these differences in methodology and sampling efforts, these data are consistent with an endemic focus that may contribute to sustaining viral circulation in nearby areas sharing similar ecological conditions.

In our study, ticks from four different genera were collected, with *H. lusitanicum* as the most abundant species (2,681/3,183 ticks). The detection of CCHFV only in *H. lusitanicum* agrees with previous surveillance studies conducted in the same region (9, 12) and supports its role in the natural maintenance of the virus, while its epidemiological role in Spain remains unclear (9, 12, 13). No *H. marginatum* pools tested positive in our study, despite being considered the main vector of CCHFV in areas where *H. lusitanicum* is absent (33). This likely reflects the relative abundance of the two species in our collections (82 pools of *H. marginatum* vs. 1,358 pools of *H. lusitanicum*), derived from the sampling strategy, which was mainly focused on wild ungulates rather than livestock or domestic animals. Notably, *H. lusitanicum* is considered a “sporadic parasite” of humans (34). An interesting hypothesis that surfaces from these observations and previous studies conducted in Spain is that *H. marginatum* may play a predominant role in human transmission, as reported in most countries of the Mediterranean regions (35, 36), while *H. lusitanicum* could be a secondary partner to circulate the virus among amplifying vertebrates hosts, which is consistent with host preferences of both species (37). No results exist for other countries in which both species of ticks coexist.

A clear predominance of *H. lusitanicum* over other tick species collected on wild ungulates, particularly red deer, was observed. Of the 1,358 *H. lusitanicum* pools, 1,122 were collected from red deer, and 44 of the 49 positive pools detected were also obtained from this host. The remaining positive pools were collected from wild boar, which usually share habitat with red deer. These findings reinforce the idea that the virus is mainly maintained between wild ungulates, complicating control efforts as managing wildlife populations is challenging.

The detection of several positive pools in the same animal (Supplementary Table 1), likely reflects the viremic state of the host or co-feeding transmission at the time of tick feeding rather than multiple independent infection events among ticks. Consequently, clusters of positive pools could be interpreted as indicators of host-level infection dynamics rather than viral prevalence in ticks.

Although CCHFV has also been detected in other tick species in Spain (8, 9, 13), none of our non-*Hyalomma* pools tested positive, probably due to their substantially lower representation in the collection (25–172 ticks, Table 2). We also analyzed CCHFV presence in questing ticks (66 pools) as a potential indicator of risk transmission to humans, but no positives were detected. This result is consistent with the generally low infection rates in unfed ticks and the reduced number of questing ticks collected in our study. To date, only one study in Spain, outside the region of concern, has detected CCHFV in questing *Hyalomma* ticks, with a positivity rate of 1.17% (7/597 pools) (9). In our study, not all the ticks feeding on the same animal were positive, which could be interpreted as the true infection rate of questing ticks feeding on an uninfected host. Molecular detection of CCHFV varied between the PCR methods used (Supplementary Table 4), likely reflecting the high genetic variability of the virus and reinforcing the need to use more than one method in molecular detection studies (38). Sequence analysis revealed the co-circulation of two genotypes in the study area: of the 44 sequenced pools, one belonged to genotype IV (Africa 4), while the remaining sequences (*n* = 43) clustered with genotype III (Africa 3). Both genotypes have also been identified in human cases in Spain (39), confirming their pathogenic relevance. In contrast, genotypes I and V, previously detected in ticks (9, 12), were not found in our study, which may reflect temporal variations in circulating strains, and the patchy nature of the microfoci.

Within genotype III, two intra-genotype sequence clusters were identified. Together with all the previously sequenced CCHFV strains from the region since 2011 (Supplementary Figure 3), these findings support the idea of high intra-genotype variability in a small geographic area, even in the same site of tick collection. Other authors, such as Negredo et al. (12), have also reported similar intra-genotype variability. Despite this nucleotide level variability, the amino acid sequence of the codified 41-residue fragment was conserved. Our phylogenetic analysis was based on a short genomic fragment, and a deeper genetic analysis on longer sequences would be necessary to better characterize the observed intra-genotype diversity.

Spain's epidemiological situation, characterized by the circulation of multiple genotypes (39, 40), contrasts with reports from other European countries, where a single genotype predominates (41–43). Geographically closer countries like France and Portugal have also detected genotype III (35, 44), suggesting the possible introduction of infected ticks via animal trade or migratory birds from Africa or Spain. Previous studies have demonstrated the role of migratory birds in the transportation of ticks from Africa to Europe (36), potentially acting as an entry pathway for new viral strains. However, we consider that the massive entry of infected ticks necessary to produce a detectable infected tick population precludes the hypothesis of migratory birds because of the low rates of parasitism by *Hyalomma*, in contrast with the thousands of larvae hatching from one single female fed on ungulates.

This study has limitations. Due to the retrospective nature of the study, sampling and analysis strategies were not consistent across years and locations; as a result, temporal and spatial patterns should be interpreted with caution and do not allow robust comparisons between years or sampling sites. The interpretation of seasonal patterns is also limited, as most ticks were collected during the hunting season, which may have influenced detection rates and host representation due to seasonal variation in tick activity. Variation of host representation across years may also have led to the inaccurate estimation of positivity rates in livestock and domestic animals. In addition, our results must also be considered under the potential biases introduced by the pooling strategy. Single-tick pools, as used in 2017, provide a more accurate estimate of infection rates, but are not always feasible due to logistic and economic factors, while pooling remains a widely used and cost-effective approach in surveillance studies (30). Because pool size was not consistently recorded, estimators based on maximum likelihood could not be applied, and the MIR is reported here only as a descriptive measure. This method assumes independence among pools, an assumption that is not met in our study when multiple pools originate from the same host and restricts the interpretation of MIR value. Despite these limitations, our multi-year study provides robust evidence of CCHFV circulation in southwestern Spain through repeated detection in multiple years and locations.

As the temporal and spatial patterns of vector-borne diseases change in Europe, surveillance studies are essential to anticipate and mitigate the risks of CCHFV emergence. Our findings highlight the need of adopting a One Health approach, integrating ecology, public health, and veterinary perspectives, in line with recent livestock studies highlighting the role of animal reservoirs in assessing human infection risk (45). The detection of CCHFV in ticks collected from wild ungulates indicates ongoing viral circulation in ecosystems shared by wildlife, domestic animals and humans, increasing the potential risk of human exposure. Serological studies in wild and domestic animals, alongside tick surveillance, are essential, especially in regions with a high density of *Hyalomma* ticks. Integrating human serological studies would further improve risk assessment. Together, these approaches provide a more comprehensive picture of CCHFV distribution and transmission risk, improving the implementation of effective prevention strategies.

## Data Availability

The nucleotide sequences obtained in this study that met the submission requirements have been deposited in GenBank (RRID:SCR_002760). The accession numbers are PX000067 - PX000070. The remaining sequences are available from the authors upon request.

## References

[1] Semper AE Olver J Warner J Cehovin A Fay PC Hart PJ . Research and product development for Crimean–Congo haemorrhagic fever: priorities for 2024–30. Lancet Infect Dis. (2024). 25:e223–34. doi: 10.1016/S1473-3099(24)00656-X 39522529

[2] Zhou Z Deng F Han N Wang H Sun S Zhang Y . Reassortment and migration analysis of Crimean-Congo haemorrhagic fever virus. J Gen Virol. (2013) 94:2536–48. doi: 10.1099/vir.0.056374-0 23939975

[3] Hawman DW Feldmann H. Crimean–Congo haemorrhagic fever virus. Nat Rev Microbiol. (2023) 21:463–77. doi: 10.1038/s41579-023-00871-9 36918725 PMC10013989

[4] Papa A Marklewitz M Paraskevopoulou S Garrison AR Alkhovsky SV Avsic-Zupanc T . History and classification of Aigai virus (formerly Crimean-Congo haemorrhagic fever virus genotype VI). J Gen Virol. (2022) 103:001734. doi: 10.1099/jgv.0.001734 35412967 PMC10026732

[5] Logan TM Linthicum KJ Bailey CL Watts DM Dohm DJ Moulton JR. Replication of Crimean-Congo hemorrhagic fever virus in four species of ixodid ticks (*Acari*) infected experimentally. J Med Entomol. (1990) 27:537–42. doi: 10.1093/jmedent/27.4.537 2117664

[6] Gonzalez JP Cornet JP Wilson ML Camicas JL. Crimean-Congo hemorrhagic fever virus replication in adult *Hyalomma truncatum* and *Amblyomma variegatum* ticks. Res Virol. (1991) 142:483–8. doi: 10.1016/0923-2516(91)90071-A 1803413

[7] Shepherd AJ Swanepoel R Shepherd SP Leman PA Mathee O. Viraemic transmission of Crimean-Congo haemorrhagic fever virus to ticks. Epidemiol Infect. (1991) 106:373–82. doi: 10.1017/S0950268800048524 1902186 PMC2272004

[8] Cuadrado-Matías R Moraga-Fernández A Peralbo-Moreno A Negredo AI Sánchez-Seco MP Ruiz-Fons F. Crimean–Congo haemorrhagic fever virus in questing non-*Hyalomma* spp. ticks in Northwest Spain, 2021. Zoonoses Public Health. (2024) 71:578–583. doi: 10.1111/zph.13130 38590023

[9] Sánchez-Seco MP Sierra MJ Estrada-Peña A Valcárcel F Molina R de Arellano ER . Widespread detection of multiple strains of Crimean-Congo hemorrhagic fever virus in ticks, Spain. Emerg Infect Dis. (2022) 28:394–402. doi: 10.3201/eid2802.211308 35076008 PMC8798670

[10] Gargili A Estrada-Peña A Spengler JR Lukashev A Nuttall PA Bente DA. The role of ticks in the maintenance and transmission of Crimean-Congo hemorrhagic fever virus: a review of published field and laboratory studies. Antivir Res. (2017) 144:93–119. doi: 10.1016/j.antiviral.2017.05.010 28579441 PMC6047067

[11] Estrada-Peña A Palomar AM Santibáñez P Sánchez N Habela MA Portillo A . Crimean-Congo hemorrhagic fever virus in ticks, Southwestern Europe, 2010. Emerg Infect Dis. (2012) 18:179–80. doi: 10.3201/eid1801.111040 22261502 PMC3310114

[12] Negredo A Habela MÁ de Arellano ER Diez F Lasala F López P . Survey of Crimean-Congo hemorrhagic fever enzootic focus, Spain, 2011–2015. Emerg Infect Dis. (2019) 25:1177–84. doi: 10.3201/eid2506.180877 31107219 PMC6537724

[13] Moraga-Fernández A Ruiz-Fons F Habela MA Royo-Hernández L Calero-Bernal R Gortazar C . Detection of new Crimean–Congo haemorrhagic fever virus genotypes in ticks feeding on deer and wild boar, Spain. Transbound Emerg Dis. (2021) 68:993–1000. doi: 10.1111/tbed.13756 32738065

[14] Palomar AM Portillo A Santibáñez S García-Álvarez L Muñoz-Sanz A Márquez FJ . Molecular (ticks) and serological (humans) study of Crimean-Congo hemorrhagic fever virus in the Iberian Peninsula, 2013–2015. Enferm Infecc Microbiol Clin. (2017) 35:344–7. doi: 10.1016/j.eimc.2017.01.009 28291670

[15] Messina J Wint W. The spatial distribution of Crimean-Congo haemorrhagic fever in Europe and neighboring areas. (2023). Available online at: https://www.ecdc.europa.eu/sites/default/files/documents/crimean-congo-haemorrhagic-fever-spatial-distribution-december-2023.pdf (Accessed July 7, 2025). doi: 10.3390/insects14090771

[16] Eslava M Carlos S Reina G. Crimean-Congo hemorrhagic fever virus: an emerging threat in Europe with a focus on epidemiology in Spain. Pathogens. (2024) 13:770. doi: 10.3390/pathogens13090770 39338961 PMC11434923

[17] ECDC. Seasonal surveillance of Crimean-Congo haemorrhagic fever (CCHF) in the EU/EEA. (2025). Available online at: https://www.ecdc.europa.eu/en/crimean-congo-haemorrhagic-fever/surveillance-and-updates/seasonal (Accessed July 7, 2025).

[18] ECDC. Historical data on local transmission of Crimean-Congo haemorrhagic fever in the EU/EEA. (2024). Available online at: https://www.ecdc.europa.eu/en/infectious-disease-topics/crimean-congo-haemorrhagic-fever/surveillance-and-updates/local-transmission-eueea-previous-years (Accessed July 7, 2025).

[19] Cuadrado-Matías R Cardoso B Sas MA García-Bocanegra I Schuster I González-Barrio D . Red deer reveal spatial risks of Crimean-Congo haemorrhagic fever virus infection. Transbound Emerg Dis. (2022) 69:630–45. doi: 10.1111/tbed.14385 34739746

[20] Baz-Flores S Herraiz C Peralbo-Moreno A Barral M Arnal MC Balseiro A . Mapping the risk of exposure to Crimean-Congo haemorrhagic fever virus in the Iberian Peninsula using Eurasian wild boar (*Sus scrofa*) as a model. Ticks Tick Borne Dis. (2024) 15:102281. doi: 10.1016/j.ttbdis.2023.102281 37995393

[21] Frías M Fischer K Castro-Scholten S Bost C Cano-Terriza D Risalde MÁ . Epidemiologic survey of Crimean-Congo hemorrhagic fever virus in suids, Spain. Emerg Infect Dis. (2024) 30:984–90. doi: 10.3201/eid3005.240074 38666621 PMC11060457

[22] GARES Project. Results of tick field studies. (2023–2024). Available online at: https://www.sanidad.gob.es/areas/alertasEmergenciasSanitarias/preparacionRespuesta/docs/Resumen_resultados_2023.pdf (Accessed July 7, 2025).

[23] EFSA Panel on Animal Health and Welfare. Scientific opinion on the role of tick vectors in the epidemiology of Crimean-Congo hemorrhagic fever and African swine fever in Eurasia. EFSA J. (2010) 8:32–3. doi: 10.2903/j.efsa.2010.1703

[24] Valcárcel F Elhachimi L Vilá M Tomassone L Sánchez M Selles SMA . Emerging *Hyalomma lusitanicum*: from identification to vectorial role and integrated control. Med Vet Entomol. (2023) 37:425–59. doi: 10.1111/mve.12660 37144688

[25] Sonenshine D. Biology of ticks. 2nd ed. Oxford: Oxford University Press (1993).

[26] Valcárcel F González J Pérez Sánchez JL Tercero Jaime JM Olmeda AS. Long-term ecological study of host-seeking adults of *Hyalomma lusitanicum* (Acari: Ixodidae) in a meso-Mediterranean climate. J Med Entomol. (2016) 53:221–4. doi: 10.1093/jme/tjv152 26477051

[27] Atkinson B Chamberlain J Logue CH Cook N Bruce C Dowall SD . Development of a real-time RT-PCR assay for the detection of Crimean-Congo hemorrhagic fever virus. Vector-Borne Zoonotic Dis. (2012) 12:786–93. doi: 10.1089/vbz.2011.0770 22217175

[28] Negredo A de la Calle-Prieto F Palencia-Herrejón E Mora-Rillo M Astray-Mochales J Sánchez-Seco MP . Autochthonous Crimean–Congo hemorrhagic fever in Spain. N Engl J Med. (2017) 377:154–61. doi: 10.1056/NEJMoa1615162 28700843

[29] Kumar S Stecher G Li M Knyaz C Tamura K MEGA X. Molecular evolutionary genetics analysis across computing platforms. Mol Biol Evol. (2018) 35:1547–9. doi: 10.1093/molbev/msy096 29722887 PMC5967553

[30] Fracasso G Grillini M Grassi L Gradoni F da Rold G Bertola M. Effective methods of estimation of pathogen prevalence in pooled ticks. Pathogens. (2023) 12:557. doi: 10.3390/pathogens12040557 37111443 PMC10146257

[31] Negredo A Sánchez-Arroyo R Díez-Fuertes F de Ory F Budiño MA Vázquez A . Fatal case of Crimean-Congo hemorrhagic fever caused by reassortant virus, Spain, 2018. Emerg Infect Dis. (2021) 27:1211–5. doi: 10.3201/eid2704.203462 33754998 PMC8007309

[32] Situation report and evaluation of the risk of transmission of the Crimean-Congo hemorrhagic fever virus (CCHFV) in Spain. (2019). Available online at: https://www.sanidad.gob.es/areas/alertasEmergenciasSanitarias/situacionRiesgo/docs/ER_FHCC.pdf (Accessed July 7, 2025).

[33] ECDC. Hyalomma marginatum - current known distribution: October 2023. (2023). Available online at: https://www.ecdc.europa.eu/en/publications-data/hyalomma-marginatum-current-known-distribution-october-2023 (Accessed July 7, 2025).

[34] Guglielmone AA Robbins RG. Hard Ticks (Acari: Ixodida: Ixodidae) Parasitizing Humans. Cham: Springer (2018). doi: 10.1007/978-3-319-95552-0

[35] Bernard C Kukla CJ Rakotoarivony I Duhayon M Stachurski F Huber K . Detection of Crimean–Congo haemorrhagic fever virus in *Hyalomma marginatum* ticks, southern France, May 2022 and April 2023. Euro Surveill. (2024) 29:2400023. doi: 10.2807/1560-7917.ES.2024.29.6.2400023 38333936 PMC10853980

[36] Palomar AM Portillo A Santibáñez P Mazuelas D Arizaga J Crespo A . Crimean-Congo hemorrhagic fever virus in ticks from migratory birds, Morocco. Emerg Infect Dis. (2013) 19:260–3. doi: 10.3201/eid1902.121193 23347801 PMC3559059

[37] Valcárcel F González J González MG Sánchez M Tercero JM Elhachimi L . Comparative Ecology of *Hyalomma lusitanicum* and *Hyalomma marginatum* Koch, 1844 (Acarina: Ixodidae). Insects. (2020) 11:30. doi: 10.3390/insects11050303 32414220 PMC7290797

[38] Gruber CEM Bartolini B Castilletti C Mirazimi A Hewson R Christova I . Geographical variability affects CCHFV detection by RT-PCR: a tool for *in-silico* evaluation of molecular assays. Viruses. (2019) 11:953. doi: 10.3390/v11100953 31623214 PMC6833031

[39] Lorenzo Juanes HM Carbonell C Sendra BF López-Bernus A Bahamonde A Orfao . Crimean-Congo hemorrhagic fever, Spain, 2013-2021. Emerg Infect Dis. (2023) 29:252–9. doi: 10.3201/eid2902.220677 36692301 PMC9881766

[40] Monsalve Arteaga L Muñoz Bellido JL Negredo AI García Criado J Vieira Lista MC Sánchez Serrano JÁ . New circulation of genotype V of Crimean-Congo haemorrhagic fever virus in humans from Spain. PLoS Negl Trop Dis. (2021) 15:e0009197. doi: 10.1371/journal.pntd.0009197 33617538 PMC7943016

[41] Emmerich P Jakupi X von Possel R Berisha L Halili B Günther S . Viral metagenomics, genetic and evolutionary characteristics of Crimean-Congo hemorrhagic fever Orthonairovirus in humans, Kosovo. Infect Genet Evol. (2018) 65:6–11. doi: 10.1016/j.meegid.2018.07.010 30006045

[42] Jakimovski D Banović P Spasovska K Rangelov G Cvetanovska M Cana F . One health investigation following a cluster of Crimean–Congo haemorrhagic fever, North Macedonia, July to November 2023. Euro Surveill. (2025) 30:2400286. doi: 10.2807/1560-7917.ES.2025.30.4.2400286 39885822 PMC11920785

[43] Leblebicioglu H Ozaras R Irmak H. Sencan, I. Crimean-Congo hemorrhagic fever in Turkey: current status and future challenges. Antiviral Res. (2016) 126:21–34. doi: 10.1016/j.antiviral.2015.12.003 26695860

[44] Zé-Zé L Nunes C Sousa M de Sousa R Gomes C Santos AS . Fatal case of Crimean-Congo hemorrhagic fever, Portugal, 2024. Emerg Infect Dis. (2025) 31:139–43. doi: 10.3201/eid3101.241264 39641460 PMC11682786

[45] Raheemi H Afsheen Z Abbas H Rizwan HM Sargison N Hamdard E . Serosurveillance of Crimean-Congo hemorrhagic fever virus antibodies in livestock as a reservoir for human infection in Afghanistan. One Health. (2025) 20:101065 doi: 10.1016/j.onehlt.2025.101065 40486752 PMC12140931

[46] Carroll SA Bird BH Rollin PE Nichol ST. Ancient common ancestry of Crimean-Congo hemorrhagic fever virus. Mol Phylogenet Evol. (2010) 55:1103–10. doi: 10.1016/j.ympev.2010.01.006 20074652

[47] Chamberlain J Cook N Lloyd G Mioulet V Tolley H Hewson R. Co-evolutionary patterns of variation in small and large RNA segments of Crimean-Congo hemorrhagic fever virus. J Gen Virol. (2005) 86:3337–41. doi: 10.1099/vir.0.81213-0 16298979

[48] Brown LD Cai TT Dasgupta A. Interval estimation for a binomial proportion. Stat Sci. (2001) 16:101–33. doi: 10.1214/ss/1009213286

