# Supplementary material for: Detection and genetic characterization of Crimean-Congo hemorrhagic fever virus in ticks from western Spain (2017, 2020-2024)
Source: Front Vet Sci. 2026 Apr 1;13:1789622. doi: 10.3389/fvets.2026.1789622 (PMC13079052; doi:10.3389/fvets.2026.1789622)
Supplement: Supplementary file 1 [file Data_Sheet_1.docx]

Supplementary Figures


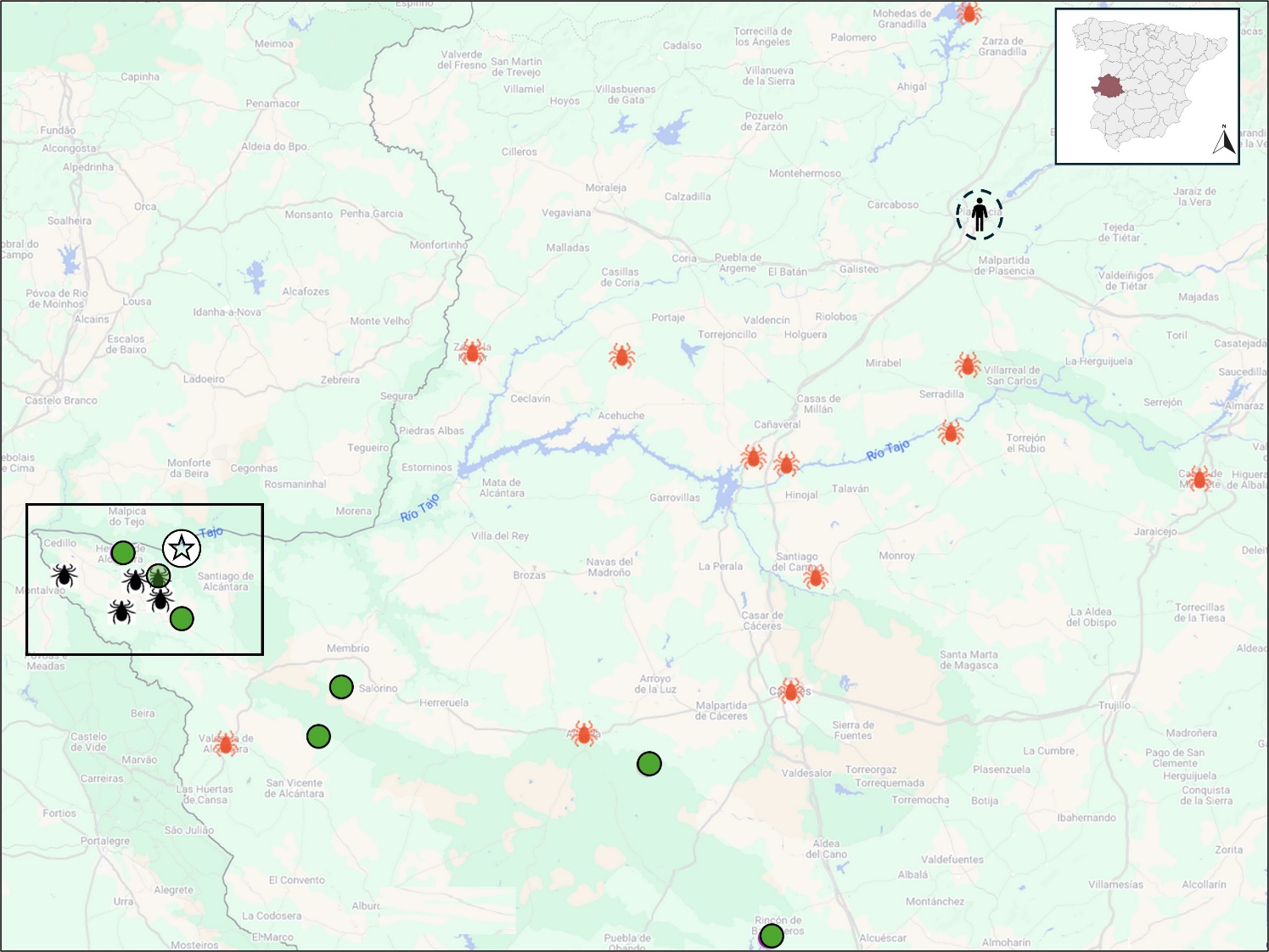


Supplementary Figure 1. Map of Cáceres province showing the only human case reported (18) in this region (circled) and all the locations where positive CCHFV ticks have been detected since 2011. Tick icons in orange indicate CCHFV-locations detected in a study conducted from 2011 to 2015 (12), while black ones correspond to a national study carried out between 2016 and 2018 (9). Sampling points from our study are marked as green circles. Marked square represents the area where CCHFV have consistently been reported since 2016, including the site of the first CCHFV detection in 2010 (11), shown as a star icon.


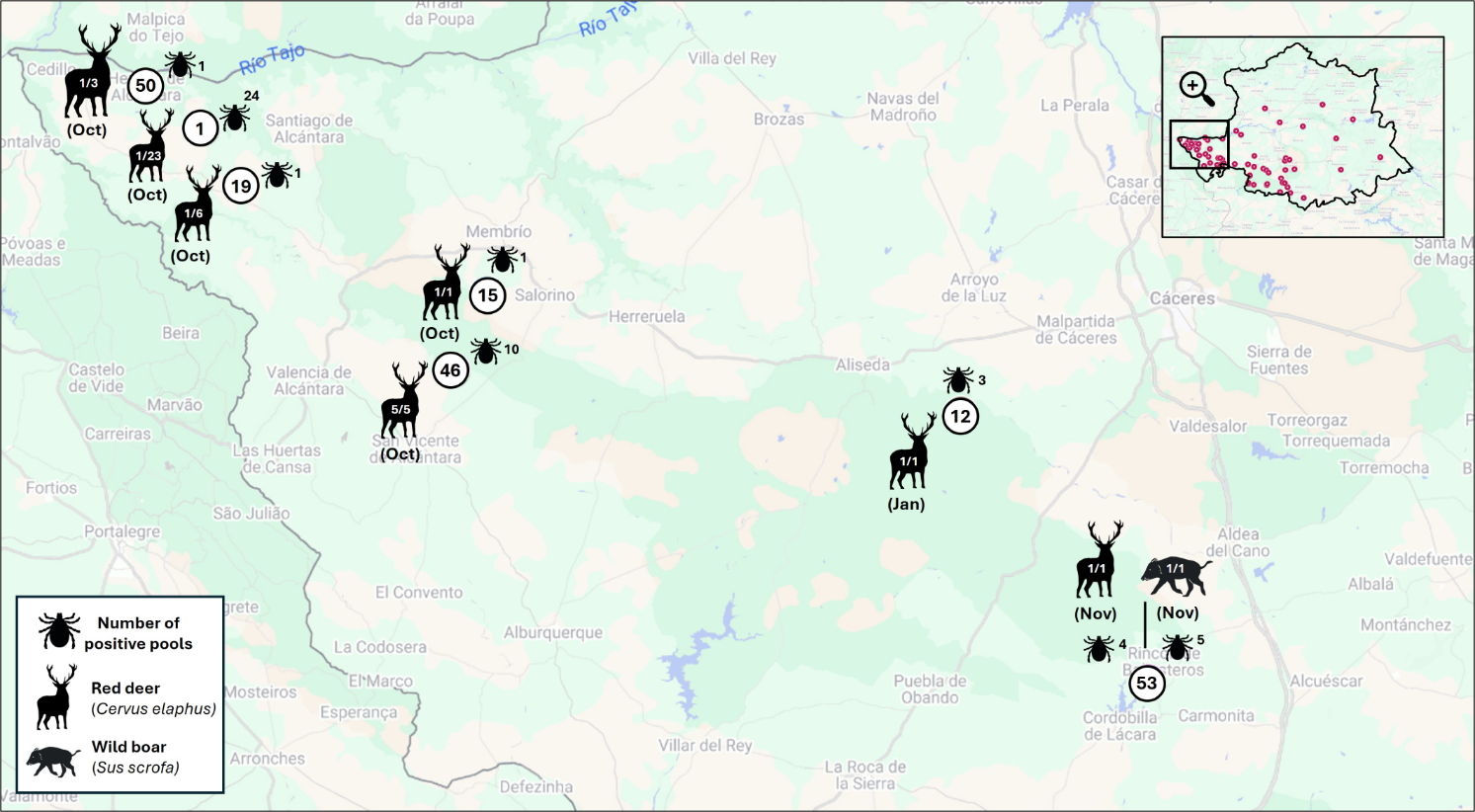


**Supplementary Figure 2.** Map of Cáceres province with a zoomed-in view of the sampling points where CCHFV-positive pools were detected, labelled with their correspondent ID numbers inside the circle. The number of positive pools is shown above the location marker. For each point, animal symbols indicate the host species in which positive pools were detected: the first value shows the number of animals with positive pools, while the value after the slash indicates the total number of that host type sampled at that point and date. The month of detection is shown below each animal symbol.


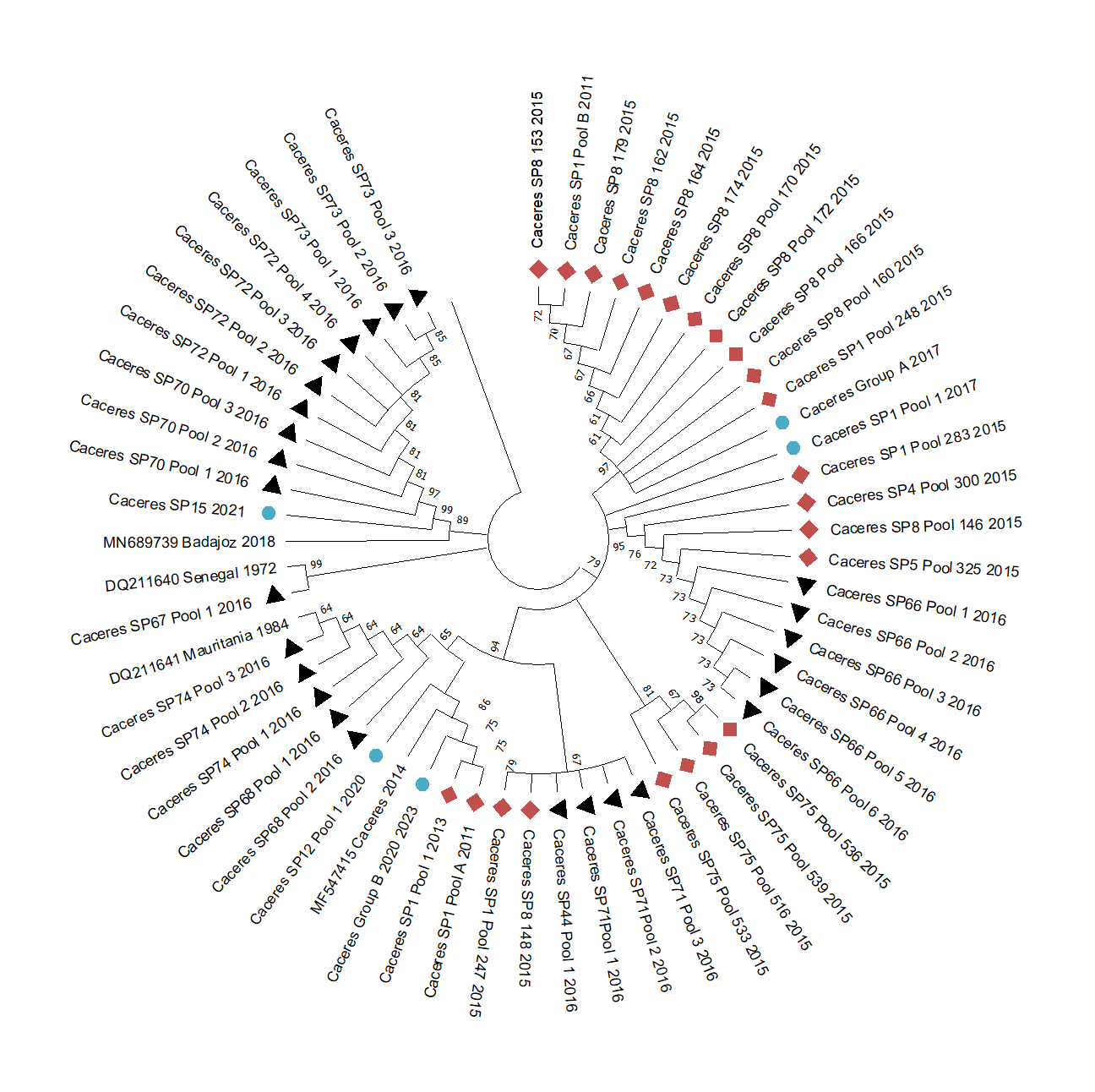


JF911699 Erve virus

**GI (Africa 1)**

**GIII (Africa 3)**

**GIV (Africa 4)**

**Supplementary Figure 3**. Phylogenetic tree with Crimean-Congo hemorrhagic fever virus sequences obtained in Cáceres since 2011. It includes our new sequenced strains detected in this study period (2017, 2020-2024) and sequences obtained in the same province in previous studies (9, 12). An additional Erve virus sequence was added as outgroup.

Blue dots indicate newly sequenced strains from this study, grouped by identical sequences, while red diamonds correspond to those strains detected in the study conducted between 2011 and 2015 by Negredo et al*.* (12). Black triangles correspond to those sequenced and analysed in a national study from 2016 to 2018 (9). All samples identified by sampling point number, pool ID and year.

The phylogenetic tree was built using the Neighbor-Joining method based on partial (127 bp) sequences of the small genomic segment of the virus. The bootstrap consensus tree inferred from 1000 replicates and values <60 are not shown. The evolutionary distances were computed using the p-distance method and are in the units of the number of base differences per site. Evolutionary analyses were conducted in MEGA X (29).

Supplementary Tables

Supplementary Table 1. Number of ticks collected by species, sampling point, and host/vegetation in Cáceres, Spain (2017, 2020–2024).

| **Year** | **ID**  **Sampling point** | **No. of ticks (No. pools)** | **No. positive pools (%)** | **Tick species** | **Host**  **(No. animals with positive pools/ total No. animals) / Vegetation** |
| --- | --- | --- | --- | --- | --- |
| 2017 | 1 | 186 (186) | 24 (12.9%) | *Hyalomma lusitanicum* | Red deer (1/23) |
|  | 2 | 60 (60) | - | *Hyalomma lusitanicum* | Red deer (0/7) |
|  |  | 20 (20) | - | *Hyalomma lusitanicum* | Wild Boar (0/2) |
|  | 3 | 13 (13) | - | *Hyalomma lusitanicum* | Red deer (0/4) |
|  |  | 24 (24) | - | *Hyalomma lusitanicum* | Fallow deer (0/4) |
|  | 4 | 77 (77) | - | *Hyalomma lusitanicum* | Red deer (0/9) |
|  |  | 4 (4) | - | *Hyalomma lusitanicum* | Wild Boar (0/1) |
|  |  | 1 (1) | - | *Dermacentor marginatus* | Wild Boar (0/1) |
|  |  | 17 (17) | - | *Hyalomma lusitanicum* | Fallow deer (0/2) |
|  | 5 | 98 (98) | - | *Hyalomma lusitanicum* | Red deer (0/7) |
|  |  | 2 (2) | - | *Hyalomma lusitanicum* | Wild Boar (0/1) |
|  | 6 | 78 (78) | - | *Hyalomma lusitanicum* | Red deer (0/6) |
|  |  | 7 (7) | - | *Hyalomma lusitanicum* | Wild Boar (0/1) |
|  | 7 | 93 (93) | - | *Hyalomma lusitanicum* | Red deer (0/10) |
|  | 8 | 88 (88) | - | *Hyalomma lusitanicum* | Red deer (0/7) |
| 2020 | 8 | 11 (4) | - | *Hyalomma lusitanicum* | Red deer (0/3) |
|  | 9 | 35 (12) | - | *Hyalomma lusitanicum* | Red deer (0/1) |
|  | 10 | 68 (23) | - | *Hyalomma lusitanicum* | Red deer (0/1) |
|  |  | 10 (3) | - | *Hyalomma lusitanicum* | Wild Boar (0/1) |
|  | 11 | 48 (16) | - | *Hyalomma lusitanicum* | Red deer (0/1) |
|  | 12 | 44 (15) | 3 (20%) | *Hyalomma lusitanicum* | Red deer (1/1) |
|  |  | 1 (1) | - | *Dermacentor marginatus* | Wild Boar (0/1) |
|  |  | 8 (3) | - | *Hyalomma lusitanicum* | Wild Boar (0/1) |
|  | 13 | 44 (14) | - | *Hyalomma lusitanicum* | Red deer (0/4) |
|  | 14 | 37 (12) | - | *Hyalomma lusitanicum* | Red deer (0/1) |
| 2021 | 5 | 6 (2) | - | *Hyalomma lusitanicum* | Wild Boar (0/1) |
|  | 13 | 16 (8) | - | *Hyalomma lusitanicum* | Red deer (0/4) |
|  |  | 2 (1) | - | *Hyalomma lusitanicum* | Wild Boar (0/1) |
|  | 15 | 1 (1) | - | *Dermacentor marginatus* | Wild Boar (0/1) |
|  |  | 24 (4) | - | *Hyalomma lusitanicum* | Wild Boar (0/1) |
|  |  | 25 (9) | 1 (9%) | *Hyalomma lusitanicum* | Red deer (1/1) |
|  | 16 | 6 (2) | - | *Hyalomma lusitanicum* | Wild Boar (0/1) |
|  |  | 23 (8) | - | *Hyalomma lusitanicum* | Red deer (0/1) |
|  | 17 | 2 (1) | - | *Hyalomma marginatum* | Bovine (0/1) |
|  |  | 34 (12) | - | *Hyalomma lusitanicum* | Bovine (0/1) |
|  | 18 | 17 (7) | - | *Hyalomma lusitanicum* | Red deer (0/3) |
|  | 19 | 8 (3) | - | *Hyalomma lusitanicum* | Red deer (0/2) |
| **Year** | **ID**  **Sampling point** | **No. of ticks (No. pools)** | **No. positive pools (%)** | **Tick species** | **Host**  **(No. animals with positive pools/ total No. animals) / Vegetation** |
| 2021 | 19 | 4 (2) | - | *Dermacentor marginatus* | Wild Boar (0/1) |
|  | 20 | 6 (3) | - | *Hyalomma lusitanicum* | Red deer (0/2) |
|  |  | 1 (1) | - | *Dermacentor marginatus* | Red deer (0/1) |
|  |  | 5 (3) | - | *Dermacentor marginatus* | Wild Boar (0/1) |
|  | 21 | 21 (8) | - | *Hyalomma lusitanicum* | Red deer (0/3) |
|  |  | 3 (1) | - | *Hyalomma lusitanicum* | Wild Boar (0/1) |
|  |  | 1 (1) | - | *Dermacentor marginatus* | Wild Boar (0/1) |
|  |  | 1 (1) | - | *Rhiphicephalus bursa* **^N^** | Red deer (0/1) |
| 2022 | 2 | 10 (5) | - | *Hyalomma lusitanicum* | Red deer (0/3) |
|  |  | 6 (3) | - | *Dermacentor marginatus* | Wild Boar (0/1) |
|  | 10 | 17 (6) | - | *Hyalomma lusitanicum* | Red deer (0/1) |
|  | 13 | 5 (2) | - | *Hyalomma lusitanicum* | Red deer (0/1) |
|  |  | 2 (1) | - | *Dermacentor marginatus* | Wild Boar (0/1) |
|  | 18 | 1 (1) | - | *Hyalomma lusitanicum* | Fallow deer (0/1) |
|  | 19 | 6 (5) | - | *Hyalomma lusitanicum* | Red deer (0/3) |
|  | 22 | 3 (3) | - | *Hyalomma marginatum* | Bovine (0/1) |
|  | 23 | 43 (15) | - | *Hyalomma lusitanicum* | Red deer (0/1) |
|  | 24 | 14 (5) | - | *Hyalomma lusitanicum* | Red deer (0/1) |
|  |  | 6 (2) | - | *Hyalomma lusitanicum* | Wild Boar (0/1) |
|  | 65 | 20 (11) | - | *Rhiphicephalus bursa* | Bovine (0/3) |
| 2023 | 2 | 26 (12) | - | *Hyalomma lusitanicum* | Red deer (0/2) |
|  | 8 | 44 (15) | - | *Hyalomma lusitanicum* | Red deer (0/7) |
|  |  | 1 (1) | - | *Hyalomma lusitanicum* **^N^** | Red deer (0/1) |
|  |  | 239 (25) | - | *Hyalomma lusitanicum* **^N^** | Vegetation |
|  |  | 12 (3) | - | *Rhiphicephalus bursa* **^N^** | Red deer (0/1) |
|  |  | 10 (1) | - | *Rhiphicephalus bursa* **^N^** | Vegetation |
|  | 9 | 14 (7) | - | *Hyalomma lusitanicum* | Wild Boar (0/3) |
|  |  | 2 (2) | - | *Dermacentor marginatus* | Wild Boar (0/1) |
|  |  | 10 (3) | - | *Hyalomma lusitanicum* | Red deer (0/1) |
|  | 10 | 120 (12) | - | *Hyalomma lusitanicum* **^N^** | Vegetation |
|  | 14 | 4 (2) | - | *Hyalomma lusitanicum* | Wild Boar (0/1) |
|  |  | 27 (8) | - | *Hyalomma lusitanicum* | Red deer (0/3) |
|  |  | 120 (12) | - | *Hyalomma lusitanicum* **^N^** | Vegetation |
|  | 15 | 34 (17) | - | *Hyalomma lusitanicum* | Red deer (0/7) |
|  | 19 | 38 (15) | 1 (6.67%) | *Hyalomma lusitanicum* | Red deer (1/6) |
|  | 21 | 8 (4) | - | *Hyalomma lusitanicum* | Red deer (0/3) |
|  | 25 | 52 (21) | - | *Rhiphicephalus bursa* | Equine (0/17) |
|  |  | 7 (3) | - | *Hyalomma marginatum* | Equine (0/2) |
|  | 26 | 80 (35) | - | *Hyalomma marginatum* | Equine (0/11) |
|  |  | 1 (1) | - | *Hyalomma marginatum* | Dog (0/1) |
|  | 27 | 21 (6) | - | *Hyalomma lusitanicum* | Bovine (0/2) |
|  |  | 12 (9) | - | *Hyalomma lusitanicum* | Ovine (0/7) |
|  |  | 5 (5) | - | *Rhiphicephalus bursa* | Ovine (0/5) |
|  | 28 | 25 (7) | - | *Rhiphicephalus bursa* | Dog (0/4) |
| **Year** | **ID**  **Sampling point** | **No. of ticks (No. pools)** | **No. positive pools (%)** | **Tick species** | **Host**  **(No. animals with positive pools/ total No. animals) / Vegetation** |
| 2023 | 28 | 1 (1) | - | *Rhiphicephalus bursa* **^N^** | Dog (0/1) |
|  |  | 62 (16) | - | *Rhiphicephalus sanguineus* | Vegetation |
|  | 29 | 7 (5) | - | *Hyalomma marginatum* | Equine (0/4) |
|  | 30 | 5 (3) | - | *Hyalomma marginatum* | Ovine (0/1) |
|  | 31 | 9 (7) | - | *Hyalomma marginatum* | Bovine (0/7) |
|  | 32 | 27 (8) | - | *Rhiphicephalus sanguineus* | Dog (0/3) |
|  |  | 12 (2) |  | *Rhiphicephalus sanguineus* **^N^** | Dog (0/1) |
|  |  | 8 (3) | - | *Hyalomma marginatum* | Equine (0/3) |
|  | 33 | 2 (2) | - | *Hyalomma marginatum* | Equine (0/1) |
|  | 34 | 50 (20) | - | *Rhiphicephalus sanguineus* | Dog (0/3) |
|  | 35 | 2 (2) | - | *Hyalomma marginatum* | Equine (0/1) |
|  | 36 | 4 (2) | - | *Hyalomma marginatum* | Equine (0/1) |
|  | 37 | 13 (5) | - | *Rhiphicephalus sanguineus* | Dog (0/2) |
|  | 38 | 3 (3) | - | *Hyalomma lusitanicum* | Red deer (0/3) |
|  |  | 1 (1) | - | *Rhiphicephalus bursa* | Red deer (0/1) |
|  | 39 | 20 (9) | - | *Hyalomma lusitanicum* | Red deer (0/5) |
|  | 40 | 4 (1) | - | *Hyalomma lusitanicum* | Red deer (0/1) |
|  |  | 16 (8) | - | *Hyalomma lusitanicum* | Wild Boar (0/3) |
|  |  | 1 (1) | - | *Hyalomma marginatum* | Wild Boar (0/1) |
|  | 41 | 23 (10) | - | *Hyalomma lusitanicum* | Red deer (0/5) |
|  | 42 | 82 (28) | - | *Hyalomma lusitanicum* | Red deer (0/8) |
|  |  | 7 (4) | - | *Hyalomma lusitanicum* | Wild Boar (0/2) |
|  |  | 1 (1) | - | *Ixodes ricinus* | Red deer (0/1) |
|  |  | 1 (1) | - | *Dermacentor marginatus* | Wild Boar (0/1) |
|  | 43 | 15 (6) | - | *Hyalomma lusitanicum* | Red deer (0/3) |
|  |  | 6 (3) | - | *Hyalomma lusitanicum* | Wild Boar (0/1) |
|  | 44 | 23 (8) | - | *Hyalomma lusitanicum* | Red deer (0/3) |
|  |  | 19 (6) | - | *Hyalomma lusitanicum* | Wild Boar (0/3) |
|  | 45 | 1 (1) | - | *Rhiphicephalus* spp*.* | Red deer (0/1) |
|  |  | 7 (2) | - | *Hyalomma lusitanicum* | Red deer (0/2) |
|  | 46 | 28 (10) | 10 (100%) | *Hyalomma lusitanicum* | Red deer (5/5) |
|  | 47 | 3 (2) | - | *Hyalomma lusitanicum* | Wild Boar (0/1) |
|  |  | 8 (5) | - | *Hyalomma lusitanicum* | Red deer (0/3) |
|  | 48 | 10 (4) | - | *Hyalomma lusitanicum* | Red deer (0/1) |
|  | 49 | 12 (5) | - | *Hyalomma lusitanicum* | Red deer (0/1) |
|  | 50 | 15 (6) | 1 (16.7%) | *Hyalomma lusitanicum* | Red deer (1/3) |
|  | 51 | 25 (10) | - | *Hyalomma lusitanicum* | Red deer (0/4) |
|  |  | 1 (1) | - | *Ixodes ricinus* | Red deer (0/1) |
|  | 52 | 18 (7) | - | *Hyalomma lusitanicum* | Red deer (0/4) |
|  | 53 | 11 (4) | 4 (100%) | *Hyalomma lusitanicum* | Red deer (1/1) |
|  |  | 12 (5) | 5 (100%) | *Hyalomma lusitanicum* | Wild Boar (1/1) |
|  | 54 | 28 (8) | - | *Hyalomma lusitanicum* | Red deer (0/4) |
|  | 55 | 1 (1) | - | *Rhiphicephalus bursa* | Red deer (0/1) |
|  |  | 1 (1) | - | *Ixodes ricinus* | Red deer (0/1) |
| **Year** | **ID**  **Sampling point** | **No. of ticks (No. pools)** | **No. positive pools (%)** | **Tick species** | **Host**  **(No. animals with positive pools/ total No. animals) / Vegetation** |
| 2023 | 56 | 17 (6) | - | *Hyalomma lusitanicum* | Red deer (0/3) |
|  |  | 1 (1) | - | *Hyalomma lusitanicum* **^N^** | Red deer (0/1) |
|  |  | 15 (5) | - | *Hyalomma lusitanicum* | Wild Boar (0/1) |
|  | 64 | 4 (1) | - | *Rhiphicephalus sanguineus* | Dog (0/1) |
|  |  | 4 (1) |  | *Rhiphicephalus sanguineus* **^N^** | Dog (0/1) |
| 2024 | 30 | 56 (20) | - | *Hyalomma lusitanicum* | Red deer (0/1) |
|  |  | 3 (1) | - | *Hyalomma lusitanicum* | Wild Boar (0/1) |
|  | 57 | 15 (7) | - | *Hyalomma lusitanicum* | Wild Boar (0/1) |
|  |  | 1 (1) | - | *Rhiphicephalus bursa* **^N^** | Wild Boar (0/1) |
|  | 58 | 30 (11) | - | *Hyalomma lusitanicum* | Wild Boar (0/2) |
|  | 59 | 10 (4) | - | *Hyalomma lusitanicum* | Red deer (0/2) |
|  |  | 2 (1) | - | *Ixodes ricinus* | Red deer (0/1) |
|  | 60 | 2 (1) | - | *Hyalomma lusitanicum* | Wild Boar (0/1) |
|  | 61 | 33 (13) | - | *Hyalomma lusitanicum* | Red deer (0/1) |
|  |  | 3 (1) | - | *Hyalomma lusitanicum* | Wild Boar (0/1) |
|  | 62 | 8 (3) | - | *Hyalomma lusitanicum* | Red deer (0/1) |
|  |  | 8 (3) | - | *Hyalomma lusitanicum* | Wild Boar (0/1) |
|  | 63 | 37 (13) | - | *Hyalomma marginatum* | Wild Boar (0/1) |
|  |  | 1 (1) | - | *Hyalomma lusitanicum* | Wild Boar (0/1) |
|  |  | 1 (1) | - | *Hyalomma marginatum* | Red deer (0/1) |
|  |  | 1 (1) | - | *Hyalomma lusitanicum* | Red deer (0/1) |
|  |  | 1 (1) | - | *Rhiphicephalus bursa* | Red deer (0/1) |

^N^ Tick in nymph stage.

Supplementary Table 2. PCR analysis of tick pools by year and method.

| **Year** | **Total No. of pools** | **Atkinson et al. (27) qRT-PCR** | **Negredo et al. (28) nested** | **Negredo et al. (28) Optimized qRT-PCR** |
| --- | --- | --- | --- | --- |
| 2017 | 768 | 768 | 768 | - |
| 2020 | 103 | 103 | 103 | - |
| 2021 | 78 | 78 | 78 | 39 |
| 2022 | 59 | 59 | 23 | 59 |
| 2023 | 478 | 478 | 58 | 478 |
| 2024 | 83 | 83 | - | 83 |
| **TOTAL** | 1,569 | 1,569 | 1,030 | 659 |

*Of the 659 pools analyzed by the optimized qRT-PCR, 120 were also analyzed with the original nested format during the methodological transition or to further characterize positive pools.

**Supplementary Table 3**. Representative groups of identical sequences used for the phylogenetic tree. Groups were defined based on identical nucleotide sequences. Each representative ID corresponds to *n* identical sequences obtained in the sampling year and point indicated in the table. Reference GenBank accession numbers, when available, are also shown.

| **Representative ID** | **Group** | **No. of sequences (*n*)** | **Sampling year** | **Sampling point** | **GenBank IDs** |
| --- | --- | --- | --- | --- | --- |
| Caceres_GroupA_2017 | A | 23 | 2017 | SP1 | - |
| Caceres_GroupB_2020, 2023 | B | 2 | 2020 | SP12 | PX000068-PX000069 |
|  |  | 1 | 2023 | SP19 | - |
|  |  | 8 | 2023 | SP46 | - |
|  |  | 7 | 2023 | SP53 | PX000067 |

**Supplementary Table 4**. Detailed results for the positive pools of ticks and their respective CCHFV genotype, according to small segment sequences.

| **Year** | **ID**  **Sampling**  **point** | **Number of pools** | **Positive pools by Atkinson et al. (27)** | **Positive pools by Negredo et al*.* (28) *** | **No. of**  **sequences** | **Genotype** |
| --- | --- | --- | --- | --- | --- | --- |
| 2017 | 1 | 186 | 15 | 24 | 24 | III |
| 2020 | 12 | 15 | 3 | 3 | 3 | III |
| 2021 | 15 | 9 | 0 | 1 | 1 | IV -Africa 4 |
| 2023 | 19 | 15 | 1 | 1 | 1 | III |
|  | 48 | 10 | 10 | 9 | 8 | III |
|  | 52 | 6 | 1 | 0 | 0 | III |
|  | 55 | 9 | 9 | 7 | 7 | III |

*This column includes results from both nested and its qRT-PCR adaptation.
